# 14. Postmarketing Safety Experience With MenACWY-TT

**DOI:** 10.1093/ofid/ofab466.216

**Published:** 2021-12-04

**Authors:** Lidia Serra, Susan Mather, Cindy Burman, Chris Webber

**Affiliations:** 1 Pfizer Inc, Collegeville, Pennsylvania; 2 Pfizer, Ltd. Hurley UK, Hurley, England, United Kingdom

## Abstract

**Background:**

MenACWY-TT (Nimenrix®), a quadrivalent meningococcal tetanus toxoid conjugate vaccine, was first licensed in 2012 and is available in 82 countries but not in the United States. MenACWY-TT is administered in infants as a 2 + 1 (6 weeks to < 6 months of age) or 1 + 1 (6 to < 12 months of age) schedule with the booster dose at 12 months of age, and from 12 months of age as a single dose. In addition to its widespread use to protect against meningococcal serogroups A, C, W, and Y, MenACWY-TT is a constituent of an investigational pentavalent meningococcal (MenABCWY) vaccine currently undergoing clinical development.

**Methods:**

Using the MenACWY-TT Periodic Safety Update Report (PSUR) with format and content in accordance with Good Pharmacovigilance Practice Module VII and International Council for Harmonisation Guideline E2C, for data up to April 19, 2020, postmarketing safety experience with MenACWY-TT is considered. The PSUR data included herein are spontaneous adverse events (AEs) from the Pfizer safety database. AEs were coded by system organ class (SOC) and preferred term (PT) using MedDRA v.22.1J.

**Results:**

The cumulative estimated exposure of MenACWY-TT was nearly 26 million doses, with the majority administered in 0- to 16-year-olds and in the Western European Union (**Figure 1**). Over the reporting period, 13,301 cumulative AEs occurred. The most common SOCs in the reporting period were general disorders and administration site conditions (n=5169; 39%); nervous system disorders (n=1986; 15%); injury, poisoning and procedural complications (n=1266; 10%); and gastrointestinal disorders (n=1031; 8%) (**Figure 2**). By PT, the most common AEs were pyrexia (n=1613; 12%), headache (n=738; 6%), and vaccination site pain (n=394; 3%) (**Figure 3**). Of the 3299 serious AEs reported, the most common were pyrexia (n=317; 10%) and headache (n=209; 6%).

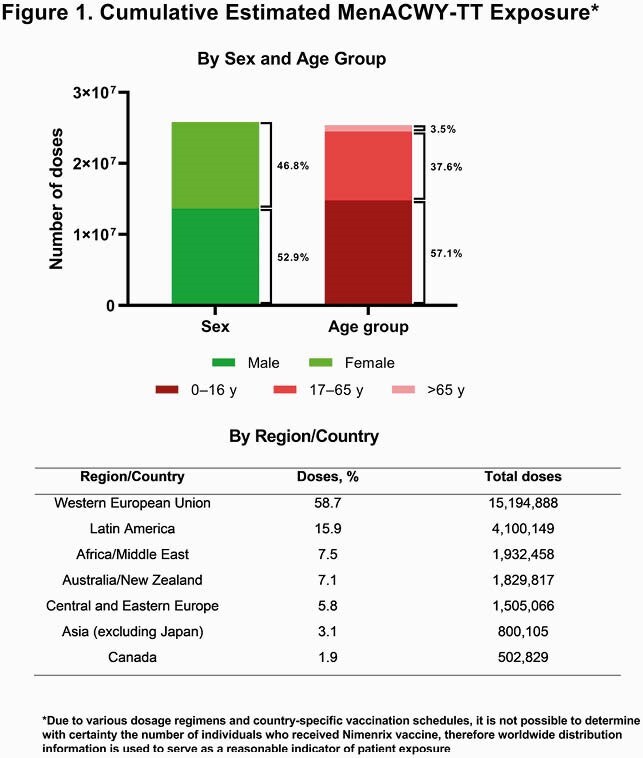

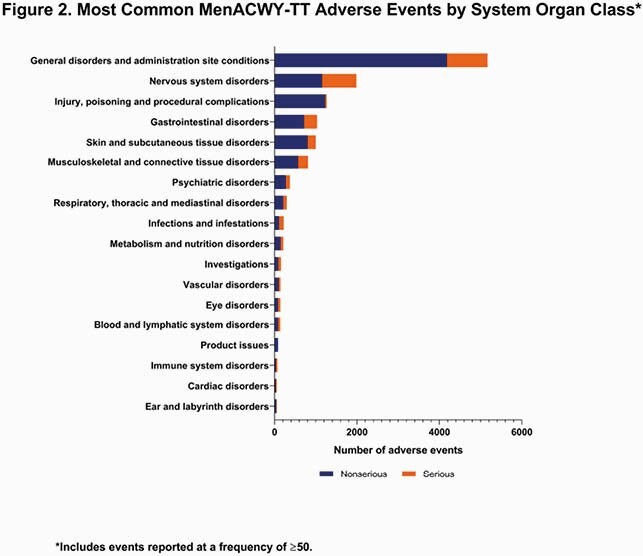

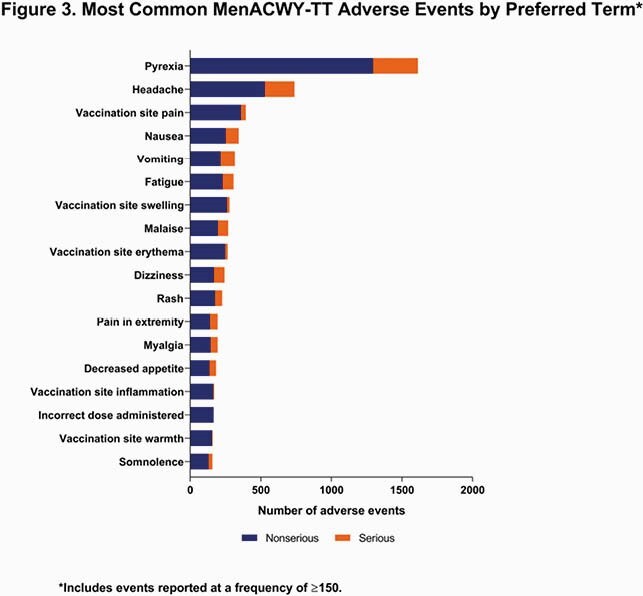

**Conclusion:**

Based on cumulative safety data in conjunction with existing efficacy and effectiveness data, the benefit-risk profile of MenACWY-TT remains favorable and is consistent with the safety profile of MenACWY-TT established in clinical studies.

**Disclosures:**

**Lidia Serra, MS**, **Pfizer Inc** (Employee, Shareholder) **Susan Mather, MD**, **Pfizer Inc** (Employee, Shareholder) **Cindy Burman, PharmD**, **Pfizer Inc** (Employee, Shareholder) **Chris Webber, MD**, **Pfizer** (Employee, Shareholder)

